# Reactive Transport Simulation of Fracture Channelization and Transmissivity Evolution

**DOI:** 10.1089/ees.2018.0244

**Published:** 2019-01-18

**Authors:** Hang Deng, Catherine A. Peters

**Affiliations:** Department of Civil and Environmental Engineering, Princeton University, Princeton, New Jersey.

**Keywords:** caprock, carbonate, channelization, fractures, geologic carbon sequestration, reactive transport

## Abstract

Underground fractures serve as flow conduits, and they may produce unwanted migration of water and other fluids in the subsurface. An example is the migration and leakage of greenhouse gases in the context of geologic carbon sequestration. This study has generated new understanding about how acids erode carbonate fracture surfaces and the positive feedback between reaction and flow. A two-dimensional reactive transport model was developed and used to investigate the extent to which geochemical factors influence fracture permeability and transmissivity evolution in carbonate rocks. The only mineral modeled as reactive is calcite, a fast-reacting mineral that is abundant in subsurface formations. The X-ray computed tomography dataset from a previous experimental study of fractured cores exposed to carbonic acid served as a testbed to benchmark the model simulation results. The model was able to capture not only erosion of fracture surfaces but also the specific phenomenon of channelization, which produces accelerating transmissivity increase. Results corroborated experimental findings that higher reactivity of the influent solution leads to strong channelization without substantial mineral dissolution. Simulations using mineral maps of calcite in a specimen of Amherstburg limestone demonstrated that mineral heterogeneity can either facilitate or suppress the development of flow channels depending on the spatial patterns of reactive mineral. In these cases, fracture transmissivity may increase rapidly, increase slowly, or stay constant, and for all these possibilities, the calcite mineral continues to dissolve. Collectively, these results illustrate that fluid chemistry and mineral spatial patterns need to be considered in predictions of reaction-induced fracture alteration and risks of fluid migration.

## Introduction

Subsurface rock formations increasingly play a role in environmental protection because they can be used for containment of greenhouse gases as in geologic carbon sequestration, for disposal of voluminous waste streams, for extraction of renewable energy as in geothermal systems, and as reliable barriers in oil and gas operations (Smith *et al.*, 2013a; Clarens and Peters, 2016; Mouzakis *et al.*, 2016; Soong *et al.*, 2018). Fractures in subsurface formations are of interest because they may enable fluid migration especially in the tight formations that serve to contain fluids and inhibit their migration (Cherubini *et al.*, 2013; Dearden *et al.*, 2013; Ramadas *et al.*, 2015; Day-Lewis *et al.*, 2017). Fluid transmissivity and solute transport in fractures are controlled by the spatial configuration of fracture apertures, that is, the fracture geometry. Mineral dissolution may alter fracture geometries if chemical disequilibrium arises from fluid perturbations (Fitts and Peters, 2013). In particular, the phenomenon of fracture channelization, which is the development of fast flow conduits after interactions with reactive fluid, has the potential to substantially increase fluid permeability and transmissivity. Channelization has been widely observed, especially in carbonate rocks (Liu *et al.*, 2005; Elkhoury *et al.*, 2013; Deng *et al.*, 2015). When reaction-induced channels are formed in fractures, the ultimate effect on fluid flow may be complicated by the effects of mechanical stresses (Ameli *et al.*, 2014), including spontaneous switching between regimes of decreasing and increasing permeability (Polak *et al.*, 2004). Improved knowledge of the development of channels in fractures is critical for understanding the evolution of hydraulic properties of deep subsurface environments.

The positive feedback between flow and reactions in fractured and porous carbonate rocks has long been understood (Ortoleva *et al.*, 1987; Dahan *et al.*, 1999; Nogues *et al.*, 2013; Smith *et al.*, 2013b; Lai *et al.*, 2014; Chen *et al.*, 2014). In order for heterogeneity in the flow field to initiate channels in fractures, surface roughness must exceed a threshold, and larger aperture heterogeneity leads to stronger channelization (Hanna and Rajaram, 1998; Szymczak and Ladd, 2009; Garcia-Rios *et al.*, 2015). In addition, channelization is controlled by flow conditions, developing primarily in flow regimes with intermediate Damköhler number and Peclet number (Detwiler *et al.*, 2003; Szymczak and Ladd, 2009; Dávila *et al.*, 2016; Menke *et al.*, 2016).

Increasingly, studies have revealed the importance of geochemistry, such as the reactivity of the fluid and the mineral composition of the rock matrix, as a controlling factor in fracture channelization. The nonlinear dependence of calcite dissolution rate on the thermodynamic driving force has long been recognized, and in earlier work, this was accounted for by adding empirical exponents to a concentration gradient (Dreybrodt, 1990; Groves and Howard, 1994; Hanna and Rajaram, 1998). The experimental studies reported in Deng *et al.* (2015) showed that dissolution patterns are also influenced by the interdependence of pH, CO_2_ content, and calcite dissolution rate. What has been studied even less is how the spatial patterns of minerals of different reactive potentials will affect fracture channelization and the subsequent evolution of hydraulic properties. Experiments have shown (Andreani *et al.*, 2008; Ellis *et al.*, 2011, 2013; Noiriel *et al.*, 2013) the importance of mineral heterogeneity in fracture geometry evolution. Modeling has shown (Deng *et al.*, 2016b) that differential dissolution creates a porous layer on the fracture surface, which may suppress flow.

Our current investigation uses reactive transport (RT) modeling to expand our understanding of geochemical processes in controlling the evolution of hydraulic properties of fractures. RT models couple the interrelated processes of flow and reaction (e.g., Kang *et al.*, 2010; Steefel *et al.*, 2015; Smith *et al.*, 2017). Sophisticated three-dimensional (3D) RT models have been used to simulate channel development in fractures, and produced very good results (Szymczak and Ladd, 2009; Starchenko *et al.*, 2016). However, they are computationally expensive, and their practical applications are limited by domain size and computational demands. As illustrated in the seminal work of Hanna and Rajaram (1998), there is the possibility of de-dimensionalization by averaging over the fracture aperture and including only fluxes in the primary dimensions of the fracture plane. Although noticeable differences have been observed between the two-dimensional (2D) and 3D models, especially after breakthrough under constant pressure gradient (Starchenko *et al.*, 2016), the 2D model is a valuable tool that is computationally practical and amenable to upscaling (Detwiler and Rajaram, 2007; Elkhoury *et al.*, 2013; Upadhyay *et al.*, 2015; Starchenko *et al.*, 2016).

In this study, we developed a 2D RT model for carbonic acid-driven reaction in fractured carbonate rock. The model employs a geochemical model that uses transition state theory, thereby capturing the dependence of reaction kinetics and thermodynamics on local concentrations of multiple species. This is particularly important in the context of carbonic acid interacting with carbonate minerals because of the common anion and the pH-dependent buffering effect. In light of the mathematical and computational complexity of coupling geochemical models with flow models, we have adopted a strategy for reducing complexity by not accounting for the diffusion limitation of mass transfer across the fracture aperture. Previous studies (Detwiler and Rajaram, 2007; Szymczak and Ladd, 2012) have made progress in this regard, modeling the transition between surface-controlled and diffusion-controlled rates, but simplified for reaction rate expressions that depend on a single variable concentration.

Another simplification adopted in this work is to consider calcite to be the only reactive mineral. The rationale for this is that calcite is unique in being very soluble, kinetically fast reacting, and often abundant enough such that its dissolution would lead to substantial volume change of void space (Fitts and Peters, 2013). Experimental evidence for the selective dissolution of calcite over other minerals, including dolomite, has been shown in fracture erosion in limestone rocks (Ellis *et al.*, 2011).

This modeling investigation benefits from having an experimental testbed to examine the performance of the 2D RT model. The intent was to determine the extent to which the simulations capture patterns of channel formation, as well as trends in reaction extent and fracture transmissivity. Because quantitative reproduction was not the purpose, all the model parameters were determined independent of the experimental dataset, and there were no fitting parameters for model calibration. The experiments investigated how carbonic acid under conditions relevant to geologic carbon storage affects fracture channelization in Indiana limestone (Deng *et al.*, 2015). Those experiments were unique in periodic X-ray computed tomography (xCT) imaging during the experiment, providing a rich dataset for comparison of model simulations.

An important finding from those experiments is that the overall calcite dissolution was lower than expected when the carbonic acid concentration was high compared to an experiment with lower carbonic acid concentration. At first glance, this is counterintuitive, but xCT imaging revealed that the high reactivity produced a deep channel through the fracture, evidence of a strong positive feedback between reaction and flow. The channel limited fracture surface accessibility to reactive fluid, explaining the low calcite dissolution, while causing the fracture transmissivity to increase rapidly, faster than what was expected, given the amount of mineral that had dissolved. The simulations presented in this study enable a theoretical examination of this phenomenon, controlling what could not be controlled in laboratory experiments, thereby isolating geochemical effects.

The model is further used in this study to examine heterogeneous mineralogy and the spatial patterns of reactive minerals, using representations of the Amherstburg limestone (Ellis and Peters, 2016), a fossiliferous wackestone, representative of carbonate rocks with a range of calcite fractions and different spatial patterns. Furthermore, the Amherstburg limestone is relevant to geologic carbon storage because it is a sedimentary rock formation that is the primary caprock of a CO_2_ injection demonstration project in Michigan.

## Methods

### 2D RT model

The governing equation of the 2D RT model is the depth-averaged advection-diffusion-reaction equation [Eq. (1)], in which “depth-averaged” refers to the dimension across the fracture aperture ${ \rm{b}}$. It is written for the total concentration, TOT_i_, of each primary component i.

\begin{align*}
 { \frac { \partial \left( { { \rm { b \;TO } } { { \rm { T } } _ { \rm { i } } } } \right) }  { \partial { \rm { t } } } } = - \nabla \cdot \left( { { \rm { \vec q \;TO } } { { \rm { T } } _ { \rm { i } } } } \right) + \nabla \cdot \left( { { \rm { b \;D } } \cdot \nabla { \rm { TO } } { { \rm { T } } _ { \rm { i } } } } \right) + { { \rm { R } } _ { \rm { i } } } \tag { 1 } 
\end{align*}

where ${ \rm{ \vec q}}$ denotes the depth-averaged volumetric flow rate and ${{ \rm{R}}_{ \rm{i}}}$ is the reaction term. Because dispersion caused by in-plane velocity variations is implicitly captured by the spatially resolved flow field, only the molecular diffusion coefficient is included in the dispersion term${ \rm{ \;D}}$, using a value of 1 × 10^−9^ m^2^/s for all species (Oelkers and Helgeson, 1988).

For this model, the primary components were chosen to be Ca^2+^, CO_3_^2−^, and H^+^. The hydrogen concentration was solved using the charge balance equation:

\begin{align*}
{{ \rm{C}}_{{{ \rm{H}}^ + }}}{ \rm{ \;}}  + 2{{ \rm{C}}_{{ \rm{C}}{{ \rm{a}}^{2 + }}}}  = {{ \rm{C}}_{{ \rm{O}}{{ \rm{H}}^ - }}}  + {{ \rm{C}}_{{ \rm{HCO}}_3^ - }}  + 2{{ \rm{C}}_{{ \rm{CO}}_3^{2 - }}} \tag{2}
\end{align*}

Table 1 includes all the reactions and the corresponding constants and parameters. The mass conservation equations for total calcium TOT_Ca_ and total carbon TOT_C_ relate the component concentrations to the primary and secondary species.

\begin{align*}
{ \rm{TO}}{{ \rm{T}}_{{ \rm{Ca}}}} = {{ \rm{C}}_{{ \rm{C}}{{ \rm{a}}^{2 + }}}} \tag{3}
\end{align*}

\begin{align*}
 { \rm { TO } } { { \rm { T } } _ { \rm { C } } } =
{ { \rm { C } } _ { { \rm { CO } } _3^ { 2 - } } } + { \frac { { {
\rm { \gamma } } _ { { { \rm { H } } ^ + } } } { { \rm { C } } _ {
{ { \rm { H } } ^ + } } } { { \rm { \gamma } } _ { { \rm { CO } }
_3^ { 2 - } } } { { \rm { C } } _ { { \rm { CO } } _3^ { 2 - } } }
}  { { { \rm { \gamma } } _ { { \rm { HCO } } _3^ - } } { { \rm {
K } } _ { { \rm { a } } 2 } } } } \\\quad + { \frac { { \rm {
\gamma } } _ { { { \rm { H } } ^ + } } ^2 { \rm { C } } _ { { {
\rm { H } } ^ + } } ^2 { { \rm { \gamma } } _ { { \rm { CO } } _3^
{ 2 - } } } { { \rm { C } } _ { { \rm { CO } } _3^ { 2 - } } } } {
{ { \rm { \gamma } } _ { { { \rm { H } } _2 } { \rm { CO } } _3^ {
\rm { * } } } } { { \rm { K } } _ { { \rm { a } } 2 } } { { \rm {
K } } _ { { \rm { a } } 1 } } } }  \tag { 4 }
\end{align*}

Embedded in Equation (4) are the mass action laws for the secondary components, in which ${{ \rm{K}}_{{ \rm{a}}1}}$ and ${{ \rm{K}}_{{ \rm{a}}2}}$ are the equilibrium constants for the dissociation of carbonic acid and bicarbonate, respectively. The activity coefficients, γ_i_, were related to ionic strength using the Davies equation.

**Table 1.** Chemical Reactions Modeled

		*log_10_K*	*k [mol/m^2^s]*
Calcite dissolution mechanisms	$CaC{O_3} \left( s \right) + {H^ + } { \mathop \Leftrightarrow \limits^{k_1}} C{a^{2 + }} + HCO_3^ -$		0.083
	$CaC{O_3} \left( s \right) + {H_2}CO_3^{*} { \mathop \Leftrightarrow \limits^{{k_2}}} C{a^{2 + }} + 2HCO_3^ -$		1.1 × 10^−4^
	$CaC{O_3} \left( s \right) { \mathop \Leftrightarrow \limits^{{k_3}}} C{a^{2 + }} + CO_3^{2 - }$	−8.48 (K_eq_)	1.5 × 10^−5^
Carbonic acid dissociation	${H_2}CO_3^{*} \Leftrightarrow {H^ + } + HCO_3^ -$	−6.35 (K_a1_)	
	$HCO_3^ - \Leftrightarrow {H^ + } + CO_3^{2 - }$	−10.33 (K_a2_)	
Water dissociation	${H_2}O \Leftrightarrow {H^ + } + O{H^ - }$	−14	

The equilibrium parameters (K) are from the PHREEQC database (Parkhurst and Appelo, 1999), and the kinetic parameters are from Deng *et al.* (2015).

The depth-averaged volumetric flow rate was calculated using the 2D local cubic law (LCL) model (e.g., Deng *et al.*, 2013), using the Reynolds equation, which is the depth-averaged approximation of the Stokes equation.

\begin{align*}
\nabla \cdot { \rm{ \vec q}} = 0 \tag{5}
\end{align*}

\begin{align*}
 { \rm { \vec q } } = - { \frac { { { \rm { b } } ^3 } { \rm { \rho g } } }  { 12 { \rm { \mu } } } } \nabla { \rm { h } } \tag { 6 } 
\end{align*}

where ${ \rm{ \mu }}$,${ \rm{ \; \rho }} ,$ ${ \rm{g}}$, and $\nabla { \rm{h}}$ are the viscosity, density, gravitation acceleration, and gradient of hydraulic head, respectively. The gravitational contribution to the hydraulic head is not included in the model.

For the single reactive mineral, there is one reaction rate, which contributes to both primary components TOT_Ca_ and TOT_C_. We assumed a kinetically controlled reaction rate using the formulation of Transition State Theory:

\begin{align*}
 { { \rm { R } } _ { { \rm { calcite } } } } = { \rm { b } } { { \rm { A } } _ { { \rm { rxn } } } } { { \rm { k } } _ { { \rm { calcite } } } } \left( { 1 - { \frac { { \rm { IAP } } }  { { { \rm { K } } _ { { \rm { eq } } } } } } } \right) / { \rm { V } } \tag { 7 } 
\end{align*}

which depends on the mineral surface area ${{ \rm{A}}_{{ \rm{rxn}}}}$. The chemical affinity term uses the ion activity product ${ \rm{IAP}}$ and the equilibrium constant for calcite dissolution (${{ \rm{K}}_{{ \rm{eq}}}}$) (Table 1). The volume of the fluid ${ \rm{V}}$ and local aperture are used to convert the reaction rate into units consistent with other terms in Equation (1). The kinetic coefficient ${{ \rm{k}}_{{ \rm{calcite}}}}$ is dependent on pH and the concentration of carbonic acid (Plummer *et al.*, 1978). This dependency is considerable and cannot be neglected under the conditions relevant to the deep subsurface. The kinetic coefficient was calculated based on the parallel reactions using Equation (8) following Chou *et al.* (1989), using parameters fitted to data measured under conditions relevant to geologic carbon storage (Deng *et al.*, 2015). The coefficient for the backward reaction ${{ \rm{k}}_{ - 3}}$ is given by ${{ \rm{k}}_3} / {{ \rm{K}}_{{ \rm{sp}}}}$.

\begin{align*}
{{ \rm{k}}_{{ \rm{calcite}}}} = {{ \rm{k}}_1}{{ \rm{a}}_{{{ \rm{H}}^ + }}}  + {{ \rm{k}}_2}{{ \rm{a}}_{{{ \rm{H}}_2}{ \rm{CO}}_3^{ \rm{*}}}}  + {{ \rm{k}}_3}{{ \rm{a}}_{{{ \rm{H}}_2}{ \rm{O}}}} - {{ \rm{k}}_{  -  3}}{{ \rm{a}}_{{ \rm{C}}{{ \rm{a}}^{2 + }}}}{{ \rm{a}}_{{ \rm{CO}}_3^{2 - }}} \tag{8}
\end{align*}

Kinetic parameters are included in Table 1.

### Numerical approach, system domain, and boundary conditions

**FIG. 1.**
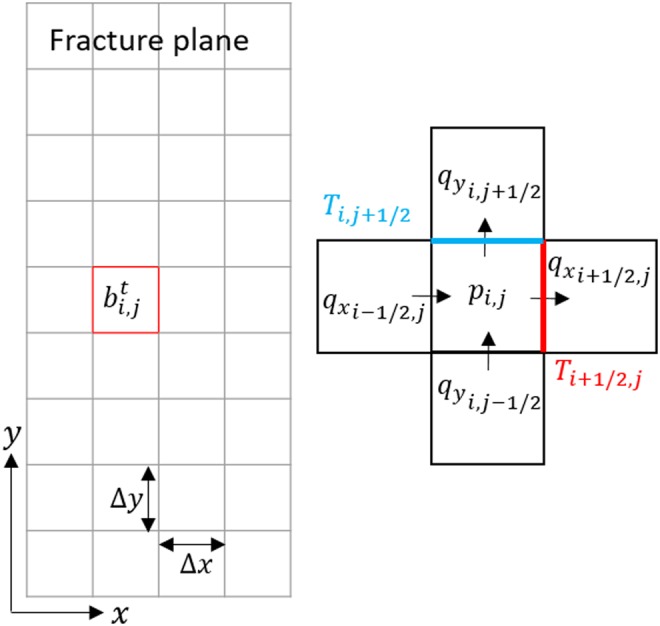
Schematic of the 2D discretization of the fracture plane. Flow is in the *y* direction. The *arrows* in the enlargement on the *right* are intended as a demonstration of the fluxes. The subscript ${i}  \pm  1 / 2$ denotes flux (*q*) or transmissivity (*T*) at the cell boundary, while pressure is given at the cell center. 2D, two dimensional.

As illustrated in Fig. 1, the fracture domain is a 2D grid parallel to the fracture plane (*x*, *y*). Each grid cell is a control volume ${ \rm{b}} \Delta { \rm{x}} \Delta { \rm{y}}$ with a dynamic aperture, ${ \rm{b}}$. The reaction surface area in each grid cell [Eq. (7)] was assumed to be the areas of the two fracture surfaces of the grid cell, that is, $2 \Delta { \rm{x}} \Delta { \rm{y}}$. The long sides of the fracture are no-flow boundaries and flow enters the fracture at the bottom and exits at the top. For all the simulations, the flow boundary conditions were selected to be a constant volumetric flow rate at the inlet and fixed pressure at the outlet. These boundary conditions are consistent with the experiments. Furthermore, this alleviates a known limitation of the 2D LCL model that when applied in fractures with large aperture variations, the model tends to overestimate flow (Brown *et al.*, 1995). By imposing a constant volumetric flow, this problem is minimized. The initial condition is that the fracture is filled with saline water with zero TOT_C_.

The governing equations were solved using a 2D explicit finite difference method. Transport and reaction equations were solved sequentially and noniteratively (Steefel and MacQuarrie, 1996) within each time step, as described in the Supplementary Material, including Supplementary Fig. S1.

### Fracture-scale effective transmissivity

Effective transmissivity, T, over the length of the fracture was used to quantify the fracture hydraulic property. As defined in Zimmerman and Bodvarsson (1996), T is the product of fracture intrinsic permeability ${{ \rm{k}}_{ \rm{f}}}$ and cross-sectional area ${{ \rm{A}}_{ \rm{C}}}$. (This definition is conceptually akin to, but not equivalent to the definition of transmissivity used in groundwater hydrology.) Here, T was inferred using Darcy's Law for flow through the entire fracture:

\begin{align*}
 { \rm { Q } } = { \frac { { \rm { T } } \,\nabla { \rm { P } } }  { \rm { \mu } } } = { \frac { { { \rm { A } } _ { \rm { C } } } { { \rm { k } } _ { \rm { f } } } \nabla { \rm { P } } }  { \rm { \mu } } } \tag { 9 } 
\end{align*}

For a given fracture geometry, transmissivity was calculated using 2D LCL flow simulations with a fixed pressure gradient along the length of the fracture $\nabla { \rm{P}}$. The total flow rate ${ \rm{Q}}$ was determined by integrating flow through all the grid cells in a given cross-section of the fracture plane. The effective transmissivity deviates from the value calculated directly from the cubic law using the geometric average aperture, and the discrepancy arises because the cubic law does not have means of accounting for roughness. Empirical relationships have been developed to estimate T by quantifying roughness using standard deviation of the apertures (Zimmerman and Bodvarsson, 1996), but the 2D LCL approach was shown to provide better estimates.

### Simulations: experimental testbed and the role of fluid chemistry

The testbed dataset was from two fractured core flow experiments that used a mineralogically homogeneous rock consisting of pure calcite (Deng *et al.*, 2015). That study investigated the effects of solution reactivity by using influents with different TOT_C_ conditions, but having effectively the same pH and state of calcite disequilibrium. To achieve the different TOT_C_ conditions, in one core flow experiment, the influent aqueous solution was equilibrated with low partial pressure of CO_2_ (*P*_CO_2__ = 12 bar [1.2 MPa]) and the other with high *P*_CO_2__ (77 bar [7.7 MPa]). (The total pressure was the same in both cases.) Both solutions had 1 mol/L of NaCl, to represent high salinity brine. The high *P*_CO_2__ influent also had 0.001 mol/L of CaCO_3_, so that both experiments were at pH 3.3. The resulting TOT_C_ concentrations were 0.30 and 1.1 M for the low and high *P*_CO_2__ cases, respectively. The resulting chemical compositions are both very far from equilibrium with respect to calcite. For both, the ratio of IAP to K_eq_ in Equation (7) is essentially zero, and the thermodynamic driving force is at its maximum. Therefore, at the fracture inlet, the rate of calcite dissolution is governed entirely by the kinetic rate limitation.

In this study, to investigate the extent to which the 2D RT model can replicate the experimental observations of mineral dissolution and transmissivity evolution, a “high TOT_C_” simulation was conducted using conditions representing the high *P*_CO_2__ experiment. To investigate the effects of influent chemistry, the comparative “low TOT_C_” simulation was conducted, representing the low *P*_CO_2__ experiment. The high and low TOT_C_ simulations were conducted with identical initial fracture geometries, isolating the effects of influent chemistry, which was not possible experimentally because the experiments were conducted with two different fractured cores.

The model system dimensions are included in Table 2. The initial fracture geometry was the fracture aperture field based on the xCT image of the unreacted fractured core in the high *P*_CO_2__ experiment, constructed using the technique of iterative local thresholding (TILT) method for 3D image segmentation (Deng *et al.*, 2016a). The high-resolution aperture map was coarsened to produce discretization with grid cell size 300 × 300 μm. The aperture value for each grid cell was determined as the arithmetic average of the corresponding pixels. The resulting geometry is called G_0_ (Table 2).

**Table 2.** Fracture Geometries and Corresponding Simulation Conditions

	*Fracture geometry*	*Fracture geometry characterization*					*Simulation conditions*		
*Fracture geometry*		*Domain size (length* × *width), cm*	*Resolution, μm*	*Average aperture, μm*	*Standard deviation, μm, G_1_, G_2_*	*Semivariogram range, μm*	*Mineral spatial distribution*	*Fluid chemistry*	*Flow rate, mL/min, Q_1_, Q_2_*
Fracture G_0_	5.49 × 2.25	300 × 300	263	150	1,800	Indiana limestone (homogeneous)	High TOT_C,_ low TOT_C_	0.5
Fractures G_1_ and G_2_	3.54 × 1.64	253 × 253	264	151, 148	1,265	REF, A, B, C	High TOT_C_	0.36, 0.18

“REF” is the case that uses homogeneous calcite; A, B, and C are the cases using different spatial patterns of calcite.

### Simulations: the role of mineral heterogeneity and spatial pattern

**FIG. 2.**
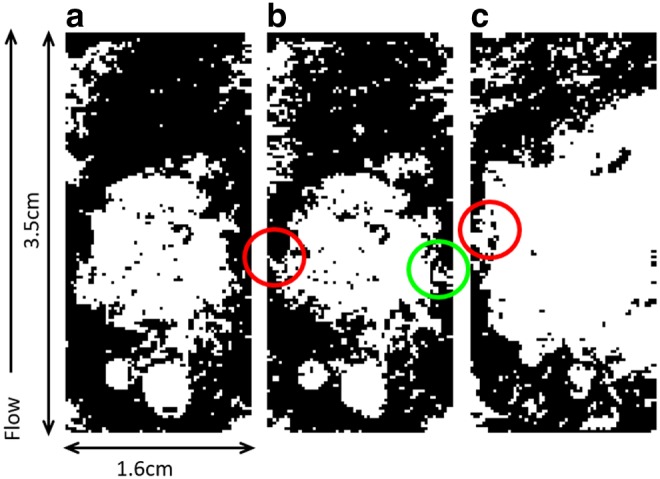
Calcite maps **(a–c)** of Amherstburg limestone. *Black* depicts calcite and *white* depicts other minerals, which are treated as nonreactive in this modeling. *Red circles* indicate areas of disconnection of calcite regions in the direction of flow along the fracture. The *green circle* highlights the narrow calcite vein.

To investigate the effects of mineral heterogeneity and spatial pattern, simulations were conducted using representations of the Amherstburg limestone. It is composed of calcite (51 wt. %), dolomite (30 wt. %), and less reactive minerals such as quartz and clay. Ellis and Peters (2016) generated binary calcite maps for the Amherstburg limestone using a novel image processing routine for 3D calcite mapping. The three fracture maps from that study were used in this work to represent scenarios of different calcite contents and spatial patterns (Fig. 2). The mineral maps (3.5 × 1.6 cm) were coarsened and have resolution of 253 μm. The resulting non-calcite grid cells were assumed to be nonreactive.

In mineral map A, calcite accounts for 62.6% of the total fracture surface, and is distributed continuously on both sides of a large unreactive blob in the center. In map B, the amount of calcite is comparable, constituting 61.7% of the fracture surface; however, the calcite is disconnected on the left, as highlighted by the red circle in Fig. 2b, and is connected by only a narrow vein on the right (green circle). Map C has the lowest amount of calcite (41.5%), which is mostly at the inlet and outlet, and along the left. Unlike A and B, in map C, calcite is disconnected as highlighted by the red circle in Fig. 2c. For reference, the simulations are compared to simulations called “REF” with similar flow and geometry conditions, but with 100% calcite.

### Other simulation conditions

Two new fracture geometries, G_1_ and G_2_, were used in the simulations of the three mineral maps and for REF. G_1_ and G_2_ are two different realizations of the same statistical properties and spatial correlation of G_0_, as shown in Table 2. Thus, the statistical nature of initial fracture apertures was a controlled variable across these simulations, something that cannot be accomplished experimentally. Comparison of the simulations from the two fracture geometries provides insights on whether differences in the random fields create significant variation in fracture alteration. If the effect is not significant, we can rule out random variability in the aperture field as a causative factor in channel formation and transmissivity evolution. For fracture G_0_, the correlation length (${ \rm{ \lambda }}$) (Supplementary Material) in both the *x* and *y* directions is ∼1,800 μm, implying that the aperture field is isotropic.

For all the heterogeneous mineral simulations, the high TOT_C_ influent chemistry was used. The volumetric flow rate, Q_1_, was scaled according to the fracture size based on the experimental flow rate, so that the flow regime is comparable to the experiment. We also studied flow rate, Q_2_, which is half the value of Q_1_ (Table 2), to demonstrate how the impacts of mineral heterogeneity may be affected by flow rate.

## Results and Discussion

### Indiana limestone high TOT_C_ simulation

**FIG. 3.**
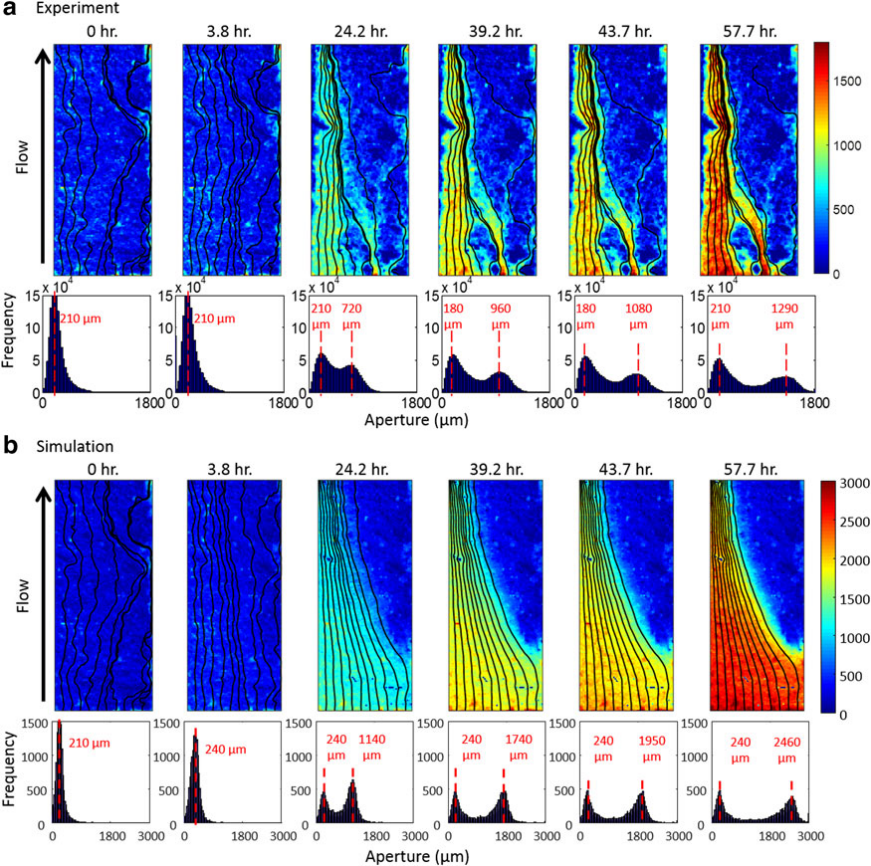
Aperture maps and aperture histograms at different simulation time points for the high TOT_C_ condition **(b)**, in comparison to the experimentally observed fractures **(a)**. The *black lines* are the streamlines representing the paths of 10 seeds released at the inlet of the fracture (along the *bottom*).

In this section, we present the results of high TOT_C_ simulation in comparison with the experimental observations (Deng *et al.*, 2015). Aperture maps were exported from the simulation at the same times as the experimental xCT scans and they are shown together with the experimental aperture maps in Fig. 3. The fracture aperture fields are in good agreement with the experimental data in regard to the emergence of the channel along the left side of the fracture starting at 24.2 h. Figure 3 also shows the resulting aperture histograms, showing the evolution from a unimodal to a bimodal distribution, which indicates the emergence of large apertures in the channel. The simulated channel is smoother than the experimental one. Factors that may have contributed to the differences in the local details of fracture geometry alteration include small-scale heterogeneities in mineralogy (MacInnis and Brantley, 1992; Levenson and Emmanuel, 2013), hydrodynamics (Szymczak and Ladd, 2009, 2012; Boon *et al.*, 2016), and pore structure (Landry and Karpyn, 2012; Selvadurai and Selvadurai, 2014). These are discussed in detail in the Supplementary Material, Supplementary Figs. S2 and S3.

**FIG. 4.**
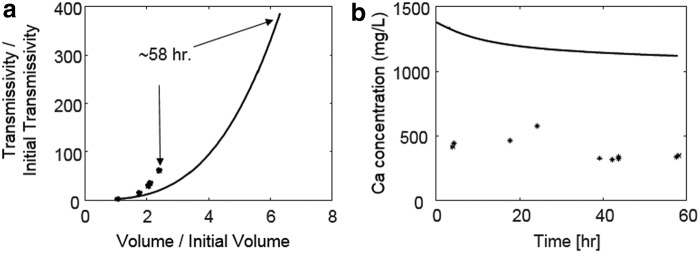
**(a)** Evolution of fracture transmissivity and fracture volume relative to their initial values, and **(b)** effluent Ca concentrations from the experimental measurements (* symbols) and the 2D RT simulations (*solid curves*). RT, reactive transport.

The effect of the channel on flow is clearly illustrated by the streamlines, which have been superimposed on the simulated aperture maps in Fig. 3. Figure 4a plots the simulated evolution of fracture transmissivity versus the fracture volume relative to their initial values. The shape of the fracture transmissivity trajectory predicted by the model shows a sharp increase as the channel deepens, which is consistent with the phenomenon of positive feedback between flow and reaction. However, the transmissivity evolution is overestimated relative to the experimental observation. This could be due to the overestimation of the overall reaction extent, which is quantified on the *x*-axis in Fig. 4a. The overestimation of reaction extent is also indicated by effluent Ca concentrations, which are approximately double those observed in the experiment (Fig. 4b). Overestimation of reaction rate has been observed in previous modeling studies, where it was attributed to the uncertainties and variability in the kinetic coefficients (Svensson and Dreybrodt, 1992; Molins *et al.*, 2014). Such discrepancy may also be caused by the 2D approach, which neglects undulation in the fracture surfaces and tends to overpredict reaction under constant pressure because the Reynolds equation overpredicts flow (Starchenko *et al.*, 2016). Also, limitations in diffusive mass transfer due to gradients in the dimension across the fracture aperture may have slowed reaction rates. To examine the significance of mass transfer in that dimension, a test simulation was performed using CrunchFlow (Steefel *et al.*, 2015), in which the lesser value of the surface reaction rate and the diffusion rate was used. The results showed that imposing a diffusion limitation does reduce the overall dissolution, but it does not affect the dissolution pattern (Supplemental Material, Supplementary Fig. S2).

Overall, the 2D RT model, without using any parameters fitted from the experimental data, corroborates that, under the experimental conditions and for a mineralogically homogeneous rock, channels form and accelerate fluid flow. The model also accurately predicts the trend of gradually decreasing effluent Ca concentration, which occurs because the development of the channel limits the reactive fluid access to an increasingly smaller area of the fracture surface. This is in support of published observations that the development of the channel reduces overall reaction (e.g., Deng *et al.*, 2015; Menke *et al.*, 2017). The model therefore provides a useful tool for exploring the research questions in this study regarding the reactive conditions that lead to channelization in fractures.

### Influent solution reactivity

**FIG. 5.**
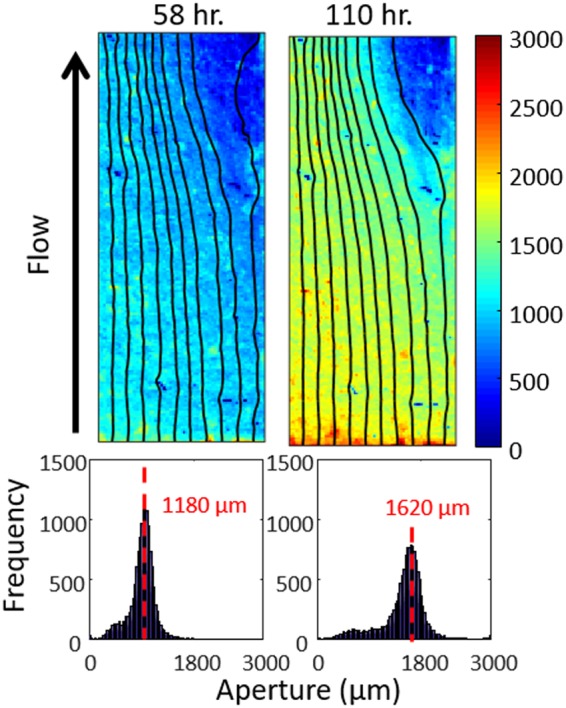
Aperture maps and aperture histograms from the low TOT_C_ simulation at h 58 and 110. The *black lines* are the streamlines, representing the paths of 10 seeds released at the inlet of the fracture (along the *bottom*).

This section presents the investigation of fluid chemistry in controlling the evolution of fracture geometry and transmissivity. For the two model simulations, the effect of the initial fracture geometry was controlled, isolating the effect of influent chemistry. The lower reactivity of the low TOT_C_ influent produces a more uniform dissolution pattern in the fracture (Fig. 5). The absence of a channel is also reflected by the unimodal aperture histograms, and the spread-out streamlines. In comparison with the decreasing effluent Ca concentrations of the high TOT_C_ simulation, the lack of channelization in the low TOT_C_ simulation produces a near steady-state reaction. The effluent Ca concentration of the low TOT_C_ simulation is mostly stabilized at ∼610 mg/L, which is lower than the high TOT_C_ simulation because of the lower reactivity of the low TOT_C_ influent (discussed extensively in Deng *et al.*, 2015). Consequently, after 58 h, the fracture volume increases by a factor of only 3.8. A long simulation (110 h) was performed for the low TOT_C_ simulation to allow the fracture to reach the same volume as the high TOT_C_ simulation, which was not possible experimentally. As illustrated by the aperture map, aperture histogram, and the streamlines, the dissolution pattern is still relatively uniform.

**FIG. 6.**
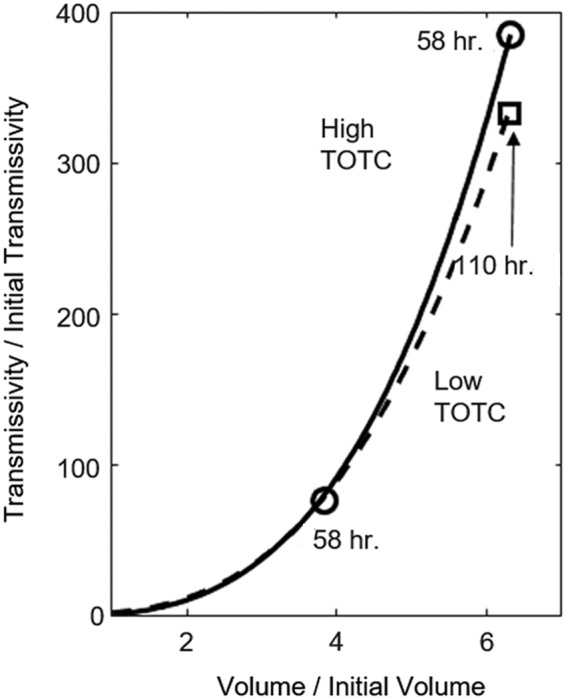
Fracture permeability evolution in relation to fracture volume increase for the low (*dashed line*) and high (*solid line*) TOT_C_ simulations. The *circles* indicate 58 h of simulation, and the *square* corresponds to the data point after 110 h in low TOT_C_ simulation.

Therefore, the numerical experiments corroborate the experimental observation and other previous findings (e.g., Liu *et al.*, 2005) that geochemical reactivity is a controlling factor of fracture channelization. The fracture transmissivity evolution in the low TOT_C_ simulation (Fig. 6) is comparable to that of the high TOT_C_ simulation, even though the reacted fracture is not channelized, but it has taken nearly twice as long to occur. It should be noted that the evolution of fracture transmissivity might be very different in the presence of geomechanical forces. Even though this is not considered in this model, examination of the change in contact area offers some insights. In this study, the grid cells with aperture smaller than the resolution of the original xCT images (30 μm) are taken as the contact points. The contact area accounts for 1.48% of the total area in the initial fracture. At the end of the high TOT_C_ simulation, dissolution has reduced the contact area to 0.71%; while in the low TOT_C_ simulation, the contact area drops to 0.30% after the same simulation time, and 0.10% after the same amount of dissolution. If subjected to normal stresses, the unchannelized portion of the fracture is likely to preserve contact points that resist fracture closure. On the contrary, the uniform enlargement in fracture apertures as observed in the low TOT_C_ simulation is more subject to fracture closure as a result of the removed contact points. Models that couple the geomechanics and RT are needed to paint a more accurate picture of the evolution of fracture hydraulic properties under confining stress, especially for cases with uniform dissolution.

### Mineral heterogeneity and dissolution pattern

**FIG. 7.**
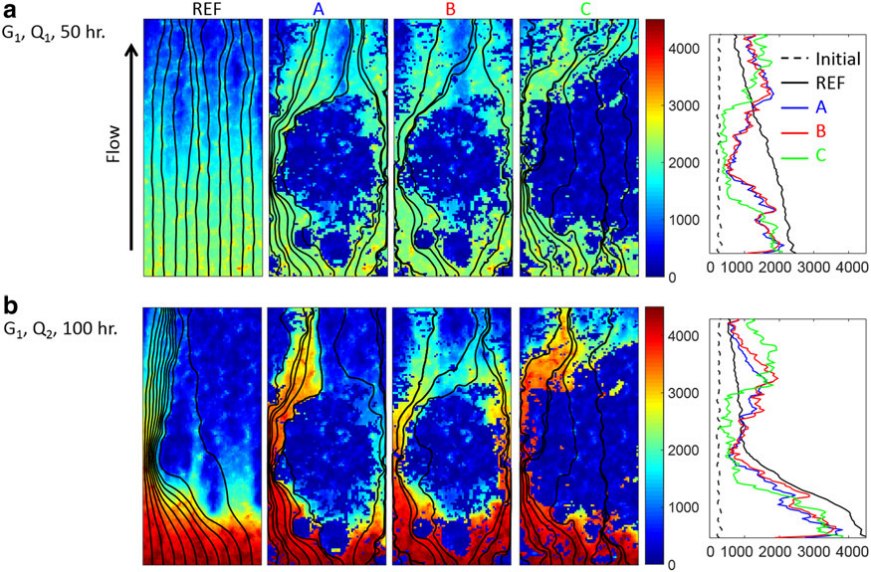
Aperture maps with streamlines and horizontally averaged aperture along the flow direction (*last column*) from the simulations of **(a)** fracture G_1_ at flow rate Q_1_ after 50 h of reaction, and **(b)** fracture G_1_ at flow rate Q_2_ after 100 h of reaction, that is, the same total flow.

In this section, we examine the case of mineralogically heterogeneous rocks and how spatial patterns of the reactive mineral affect fracture channelization and transmissivity evolution. The reference case, REF, for which the entire fracture surface is calcite, serves as a comparative baseline because channelization is solely due to aperture variations in the initial fracture geometry. As shown in Fig. 7b, for the REF simulations with the lower flow rate (Q_2_) a single channel developed, whereas with the higher flow rate (Q_1_) (Fig. 7a), the streamlines indicate fairly uniform aperture increase over the width of the fracture, with a slight hint of channels starting to emerge.

For maps A, B, and C, the dissolution patterns are additionally affected by the spatial distributions of the reactive mineral. The apertures located within the nonreactive blob remain unchanged, and thus over time, preferential flow paths emerge around it. At the high flow rate of Q_1_ (Fig. 7a), the spatial pattern observed in REF is not sustained. This implies that any initial perturbation caused by the fracture geometry is ultimately outcompeted by the variations in local reactivity caused by mineral heterogeneity. At the low flow rate (Q_2_) (Fig. 7b), the underlying fracture geometry does still have an effect. For instance, with mineral map A, there is calcite along both the right and left sides, but a channel forms only on the left side because that is where the initial fracture geometry favors channel development. The aperture increase on the right side is limited because that area does not coincide with where the initial aperture field would favor channel development.

Mineral map B is an example of a case in which the location of the reactive mineral and where the channel would form based on the underlying geometry are spatially out of sync. The positive feedback between flow and reaction is delayed, but eventually it takes hold on the right side where there is a continuous path of calcite dissolution creating a continuous channel to attract more flow of reactive fluid. In the simulations of mineral map C, the formation of the channel is suppressed as calcite is disconnected along the flow direction.

### Mineral heterogeneity and transmissivity evolution

**FIG. 8.**
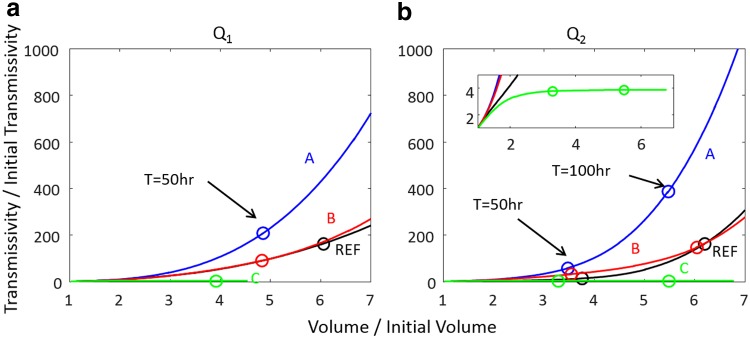
Transmissivity increase in relation to fracture volume increase for different mineral spatial patterns and flow rates. These simulation results are for fractures with geometry G_1_. The *circles* highlight the prediction values at h 50 for flow rate Q_1_, (**a**) and h 100 for flow rate Q_2_ (**b**).

Figure 8 shows the simulated evolution of fracture transmissivity versus the fracture volume relative to initial values for all the heterogeneous mineral simulations. In all cases, the overall aperture increase, that is, fracture volume increase, is the highest for REF. However, the transmissivity increases for the heterogeneous cases are sometimes larger and sometimes smaller than REF.

The increases in transmissivity for the fractures with mineral spatial pattern A are most rapid. This is because the spatial pattern of calcite is continuous along the flow direction, and allows a continuous flow channel to develop. This is especially true for flow rate Q_2_. In mineral map B, there is about the same amount of calcite as in map A, but the calcite is less connected and calcite dissolution tends to substantially increase fracture roughness. The consequent transmissivity increase is moderate compared with mineral map A. This is consistent with previous studies (e.g., Deng *et al.*, 2013), which showed that an increase in fracture roughness results in a lower fracture permeability and thus transmissivity in comparison with a parallel-wall fracture of equivalent aperture.

With mineral map C, the fracture transmissivity is predicted to increase and then remain constant. Initially, dissolution enhances flow in the calcite regions. Then, the strictures of nonreactive mineral emerge as dissolution continues to enlarge fracture apertures where there is calcite. Eventually, the persistent strictures, evident on the plot of average aperture along the flow direction (Fig. 7), limit the flow. Fracture transmissivity levels off, and ongoing calcite dissolution no longer contributes to flow enhancement.

The aperture maps and transmissivity trajectories for simulations using fracture geometry G_2_ are included in Supplementary Materials, Supplementary Figs. S4 and S5. Comparing the simulations for the two fracture geometries, G_1_ and G_2_, shows minimal effects arising from randomly generated realizations of statistically identical sets of apertures. This gives us confidence that the effects of different reactive mineral patterns (REF, A, B, and C) and the effects of flow rate (Q_1_ and Q_2_) are not random manifestations.

Although these simulations are performed on fractures on the scale of centimeters, the findings provide important insights for larger systems. Along the flow direction, the channelization may be hindered by mineral spatial heterogeneity, but the reactivity of the fluid can be sustained and penetrates further downstream, therefore increasing the possibility of breakthrough of a channel or reducing the breakthrough time. Regardless of the length of the fracture, the presence of discontinuity even at small scales will suppress the formation of a channel. However, if the fracture is extended along the lateral direction, the likelihood of finding a path that has continuous reactive mineral increases, and thus the possibility of suppressing flow channeling decreases.

## Conclusions

In this study, a 2D RT model was developed to describe mineral reactions on fracture surfaces and the resulting changes in the fracture transmissivity (which is related to permeability). This 2D RT model incorporates a multispecies geochemical model with a mineral dissolution rate equation based on transition state theory and the coupling of parallel catalyzed reactions applicable to high pressure carbonic acid conditions. This work also benefits from the ability to compare simulation results with experimental results, in which the effect of geochemical conditions was examined using temporal xCT imaging. Comparison of the simulation results and previous experimental results confirmed that a 2D model is able to capture the process of channelization in fractures and the rapid evolution of fracture transmissivity. The model overestimates reaction extent (by a factor of 2) and underestimates the evolution of transmissivity due to model simplifications and parameter uncertainties, but the model is a valuable tool for exploring geochemical conditions that promote or inhibit channelization.

In this study, we used the 2D model to investigate how channelization is influenced by fluid chemistry. In a mineralogically homogeneous rock, the positive feedback between flow of reactive fluid and mineral dissolution can produce channelization, which leads to rapid acceleration of transmissivity evolution. The simulation results show that the reactivity of the influent chemistry affects the strength of channelization, corroborating previous experimental observations. While the influent with higher reactivity leads to stronger channelization, the influent with lower reactivity results in more uniform dissolution.

One new contribution of our study is the consideration of spatial pattern of the reactive mineral in mineralogically heterogeneous rock and its impacts on fracture channelization. The numerical experiments using the Amherstburg limestone calcite maps demonstrated that the spatial patterns of the reactive mineral can largely affect the spatial patterns of fracture aperture change, and control fracture transmissivity evolution by facilitating or suppressing the development of flow channels. If the reactive mineral is continuous along the flow direction, formation of flow channels is facilitated and the fracture transmissivity increases rapidly. If the reactive mineral is less well connected in the direction of flow, the increase in transmissivity resulting from channelization is limited. If the reactive mineral is disconnected, the formation of flow channels is suppressed, and fracture transmissivity does not increase in response to the dissolution and aperture increase, because of the preserved flow strictures imposed by the unreactive mineral. When the continuous reactive mineral overlaps with the channel that would develop without mineral heterogeneity, whose location is largely controlled by the underlying fracture geometry, the process of channelization and subsequent fracture transmissivity evolution are further amplified. In contrast, if the spatial pattern of the reactive mineral and the preferential flow paths determined by the geometry do not overlap, channelization associated with mineral heterogeneity will be dampened.

## Supplementary Material

Supplemental data

## References

[B1] Ameli P., Elkhoury J., Morris J., and Detwiler R. (2014). Fracture permeability alteration due to chemical and mechanical processes: A coupled high-resolution model. Rock Mech. Rock Eng. 47, 1563

[B2] Andreani M., Gouze P., Luquot L., and Jouanna P. (2008). Changes in seal capacity of fractured claystone caprocks induced by dissolved and gaseous CO_2_ seepage. Geophys. Res. Lett. 35, L14404

[B3] Boon M., Bijeljic B., Niu B., and Krevor S. (2016). Observations of 3-D transverse dispersion and dilution in natural consolidated rock by X-ray tomography. Adv. Water Resour. 96, 266

[B4] Brown S.R., Stockman H.W., and Reeves S.J. (1995). Applicability of the Reynolds equation for modeling fluid flow between rough surfaces. Geophys. Res. Lett. 22, 2537

[B5] Chen L., Kang Q., Viswanathan H.S., and Tao W. (2014). Pore-scale study of dissolution-induced changes in hydrologic properties of rocks with binary minerals. Water Resour. Res. 50, 9343

[B6] Cherubini C., Giasi C., and Pastore N. (2013). Fluid flow modeling of a coastal fractured karstic aquifer by means of a lumped parameter approach. Environ. Earth Sci. 70, 2055

[B7] Chou L., Garrels R.M., and Wollast R. (1989). Comparative-study of the kinetics and mechanisms of dissolution of carbonate minerals. Chem. Geol. 78, 269

[B8] Clarens A.F., and Peters C.A. (2016). Mitigating climate change at the carbon water nexus: A call to action for the environmental engineering community. Environ. Eng. Sci. 33, 719 28031695 10.1089/ees.2016.0455 PMC5160138

[B9] Dahan O., Nativ R., Adar E., Berkowitz B., and Ronen Z. (1999). Field observation of flow in a fracture intersecting unsaturated chalk. Water Resour. Res. 35, 3315

[B10] Dávila G., Luquot L., Soler J.M., and Cama J. (2016). Interaction between a fractured marl caprock and CO_2_-rich sulfate solution under supercritical CO_2_ conditions. Int. J. Greenh. Gas Con. 48, 105

[B11] Day-Lewis F.D., Slater L.D., Robinson J., Johnson C.D., Terry N., and Werkema D. (2017). An overview of geophysical technologies appropriate for characterization and monitoring at fractured-rock sites. J. Environ. Manage. 204, 709 28434821 10.1016/j.jenvman.2017.04.033 PMC5894821

[B12] Dearden R.A., Noy D.J., Lelliott M.R., Wilson R., and Wealthall G.P. (2013). Release of contaminants from a heterogeneously fractured low permeability unit underlying a DNAPL source zone. J. Contam. Hydrol. 153, 141 24119249 10.1016/j.jconhyd.2011.05.006

[B13] Deng H., Ellis B.R., Peters C.A., Fitts J.P., Crandall D., and Bromhal G.S. (2013). Modifications of carbonate fracture hydrodynamic properties by CO_2_-acidified brine flow. Energy Fuels 27, 4221

[B14] Deng H., Fitts J.P., Crandall D., McIntyre D., and Peters C.A. (2015). Alterations of fractures in carbonate rocks by CO_2_-acidified brines. Environ. Sci. Technol. 49, 10226 26205851 10.1021/acs.est.5b01980

[B15] Deng H., Fitts J.P., and Peters C.A. (2016a). Fracture characterization from X-ray CT imaging—Introduction of the technique of iterative local thresholding (TILT). Computat. Geosci. 20, 231

[B16] Deng H., Molins S., Steefel C., DePaolo D., Voltolini M., Yang L., and Ajo-Franklin J. (2016b). A 2.5D reactive transport model for fracture alteration simulation. Environ. Sci. Technol. 50, 7564 27357572 10.1021/acs.est.6b02184

[B17] Detwiler R., Glass R., and Bourcier W. (2003). Experimental observations of fracture dissolution: The role of Peclet number on evolving aperture variability. Geophys. Res. Lett. 30, 1648

[B18] Detwiler R., and Rajaram H. (2007). Predicting dissolution patterns in variable aperture fractures: Evaluation of an enhanced depth-averaged computational model. Water Resour. Res. 43, W04403

[B19] Dreybrodt W. (1990). The role of dissolution kinetics in the development of karst aquifers in limestone: A model simulation of karst evolution. J. Geol. 98, 639

[B20] Elkhoury J.E., Ameli P., and Detwiler R.L. (2013). Dissolution and deformation in fractured carbonates caused by flow of CO_2_-rich brine under reservoir conditions. Int. J. Greenh. Gas Con. 16 (Suppl. 1), S203

[B21] Ellis B.R., Fitts J.P., Bromhal G.S., McIntyre D.L., Tappero R., and Peters C.A. (2013). Dissolution-driven permeability reduction of a fractured carbonate caprock. Environ. Eng. Sci. 30, 187 23633894 10.1089/ees.2012.0337 PMC3636598

[B22] Ellis B.R., and Peters C.A. (2016). 3D mapping of calcite and a demonstration of its relevance to permeability evolution in reactive fractures. Adv. Water Resour. 95, 246

[B23] Ellis B.R., Peters C.A., Fitts J.P., Bromhal G.S., McIntyre D.L., Warzinski R.P., and Rosenbaum E. (2011). Deterioration of a fractured carbonate caprock exposed to CO_2_-acidified brine flow. Greenh. Gases Sci. Technol. 1, 248

[B24] Fitts J.P., and Peters C.A. (2013). Caprock fracture dissolution and CO_2_ leakage. Rev. Mineral. Geochem. 77, 459

[B25] Garcia-Rios M., Luquot L., Soler J.M., and Cama J. (2015). Influence of the flow rate on dissolution and precipitation features during percolation of CO_2_-rich sulfate solutions through fractured limestone samples. Chem. Geol. 414, 95

[B26] Groves C.G., and Howard A.D. (1994). Early development of karst systems: 1. Preferential flow path enlargement under laminar flow. Water Resour. Res. 30, 2837

[B27] Hanna R.B., and Rajaram H. (1998). Influence of aperture variability on dissolutional growth of fissures in Karst formations. Water Resour. Res. 34, 2843

[B28] Kang Q., Lichtner P.C., Viswanathan H.S., and Abdel-Fattah A.I. (2010). Pore scale modeling of reactive transport involved in geologic CO_2_ sequestration. Transp. Porous Media 82, 197

[B29] Lai K.-H., Chen J.-S., Liu C.-W., and Yang S.-Y. (2014). Effect of permeability-porosity functions on simulated morphological evolution of a chemical dissolution front. Hydrol. Process. 28, 16

[B30] Landry C.J., and Karpyn Z.T. (2012). Single-phase lattice Boltzmann simulations of pore-scale flow in fractured permeable media. Int. J. Oil Gas Coal Technol. 5, 182

[B31] Levenson Y., and Emmanuel S. (2013). Pore-scale heterogeneous reaction rates on a dissolving limestone surface. Geochim. Cosmochim. Acta 119, 188

[B32] Liu J.S., Polak A., Elsworth D., and Grader A. (2005). Dissolution-induced preferential flow in a limestone fracture. J. Contam. Hydrol. 78, 53 15936847 10.1016/j.jconhyd.2005.03.001

[B33] MacInnis I.N., and Brantley S.L. (1992). The role of dislocations and surface morphology in calcite dissolution. Geochim. Cosmochim. Acta 56, 1113

[B34] Menke H.P., Andrew M.G., Blunt M.J., and Bijeljic B. (2016). Reservoir condition imaging of reactive transport in heterogeneous carbonates using fast synchrotron tomography—Effect of initial pore structure and flow conditions. Chem. Geol. 428, 15

[B35] Menke H.P., Bijeljic B., and Blunt M.J. (2017). Dynamic reservoir-condition microtomography of reactive transport in complex carbonates: Effect of initial pore structure and initial brine pH. Geochim. Cosmochim. Acta 204, 267

[B36] Molins S., Trebotich D., Yang L., Ajo-Franklin J.B., Ligocki T.J., Shen C., and Steefel C.I. (2014). Pore-scale controls on calcite dissolution rates from flow-through laboratory and numerical experiments. Environ. Sci. Technol. 48, 7453 24865463 10.1021/es5013438

[B37] Mouzakis K.M., Navarre-Sitchler A.K., Rother G., Bañuelos J.L., Wang X., Kaszuba J.P., Heath J.E., Miller Q.R.S., Alvarado V., and McCray J.E. (2016). Experimental study of porosity changes in shale caprocks exposed to CO_2_-saturated brines I: Evolution of mineralogy, pore connectivity, pore size distribution, and surface area. Environ. Eng. Sci. 33, 725

[B38] Nogues J.P., Fitts J.P., Celia M.A., and Peters C.A. (2013). Permeability evolution due to dissolution and precipitation of carbonates using reactive transport modeling in pore networks. Water Resour. Res. 49, 6006

[B39] Noiriel C., Gouze P., and Made B. (2013). 3D analysis of geometry and flow changes in a limestone fracture during dissolution. J. Hydrol. 486, 211

[B40] Oelkers E.H., and Helgeson H.C. (1988). Calculation of the thermodynamic and transport properties of aqueous species at high pressures and temperatures: Aqueous tracer diffusion coefficients of ions to 1000°C and 5 kb. Geochim. Cosmochim. Acta 52, 63

[B41] Ortoleva P., Merino E., Moore C., and Chadam J. (1987). Geochemical self-organization. 1. Reaction-transport feedbacks and modeling approach. Am. J. Sci. 287, 979

[B42] Parkhurst D.L., and Appelo C.A.J. (1999). User's guide to PHREEQC (version 2): A computer program for speciation, batch-reaction, one-dimensional transport, and inverse geochemical calculations. U.S. Geological Survey Water-Resources Investigations Report 99-4259

[B43] Plummer L.N., Wigley T.M.L., and Parkhurst D.L. (1978). Kinetics of calcite dissolution in CO_2_-water systems at 5 degree to 60 degree C and 0. 0 to 1. 0 atm CO_2_ Am. J. Sci. 278, 179

[B44] Polak A., Elsworth D., Liu J., and Grader A.S. (2004). Spontaneous switching of permeability changes in a limestone fracture with net dissolution. Water Resour. Res. 40, W03502

[B45] Ramadas M., Ojha R., and Govindaraju R.S. (2015). Current and future challenges in groundwater. II: Water quality modeling. J. Hydrol. Eng. 20, A4014008

[B46] Selvadurai P.A., and Selvadurai A.P.S. (2014). On the effective permeability of a heterogeneous porous medium: The role of the geometric mean. Philos. Mag. 94, 2318

[B47] Smith M.M., Hao Y., and Carroll S.A. (2017). Development and calibration of a reactive transport model for carbonate reservoir porosity and permeability changes based on CO_2_ core-flood experiments. Int. J. Greenh. Gas Con. 57, 73

[B48] Smith M.M., Sholokhova Y., Hao Y., and Carroll S.A. (2013a). Evaporite caprock integrity: An experimental study of reactive mineralogy and pore-scale heterogeneity during brine-CO_2_ exposure. Environ. Sci. Technol. 47, 262 22831758 10.1021/es3012723

[B49] Smith M.M., Sholokhova Y., Hao Y., and Carroll S.A. (2013b). CO_2_-induced dissolution of low permeability carbonates. Part I: Characterization and experiments. Adv. Water Resour. 62, 370

[B50] Soong Y., Crandall D., Howard B.H., Haljasmaa I., Dalton L.E., Zhang L., Lin R., Dilmore R.M., Zhang W., Shi F., and Mclendon T.R. (2018). Permeability and mineral composition evolution of primary seal and reservoir rocks in geologic carbon storage conditions. Environ. Eng. Sci. 35, 391

[B51] Starchenko V., Marra C.J., and Ladd A.J.C. (2016). Three-dimensional simulations of fracture dissolution. J. Geophys. Res. Solid Earth 121, 6421

[B52] Steefel C.I., Appelo C.A.J., Arora B., Jacques D., Kalbacher T., Kolditz O., Lagneau V., Lichtner P.C., Mayer K.U., Meeussen J.C.L., Molins S., Moulton D., Shao H., Simunek J., Spycher N., Yabusaki S.B., and Yeh G.T. (2015). Reactive transport codes for subsurface environmental simulation. Computat. Geosci. 19, 445

[B53] Steefel C.I., and MacQuarrie K.T.B. (1996). Approaches to modeling of reactive transport in porous media. Rev. Mineral. Geochem. 34, 83

[B54] Svensson U., and Dreybrodt W. (1992). Dissolution kinetics of natural calcite minerals in CO_2_-water systems approaching calcite equilibrium. Chem. Geol. 100, 129

[B55] Szymczak P., and Ladd A. (2009). Wormhole formation in dissolving fractures. J. Geophys. Res. 114, B06203

[B56] Szymczak P., and Ladd A.J.C. (2012). Reactive-infiltration instabilities in rocks: Fracture dissolution. J. Fluid Mech. 702, 239

[B57] Upadhyay V.K., Szymczak P., and Ladd A.J.C. (2015). Initial conditions or emergence: What determines dissolution patterns in rough fractures? *J*. Geophys. Res. Solid Earth. 120, 6102

[B58] Zimmerman R., and Bodvarsson G. (1996). Hydraulic conductivity of rock fractures. Transp. Porous Media 23, 1

