# Supplementary material for: Reactive Transport Simulation of Fracture Channelization and Transmissivity Evolution
Source: Environ Eng Sci. 2019 Jan 18;36(1):90–101. doi: 10.1089/ees.2018.0244 (PMC6354614; doi:10.1089/ees.2018.0244)
Supplement: Supplemental data [file Supp_Data.pdf]

Environmental Engineering Science

Supplemental Material for

Reactive Transport Simulation of Fracture Channelization and Transmissivity Evolution

Hang Deng and Catherine A. Peters*

Department of Civil & Environmental Engineering
Princeton University
Princeton NJ 08540 U.S.A.
cap@princeton.edu, 1-609-258-5645

Contents:

1. Numerical approach used in the model;
2. Spatial correlation length of aperture field;
3. Potential explanations of the discrepancy between the high TOT_C simulation and experiment.
4. Simulation results for the G2 fracture geometry.

1. Numerical approach

The concentrations of TOTCa and TOTC for each time step are solved using the sequentially non-iterative method [*Steefel and MacQuarrie*, 1996]. It means that each single time step consists of two steps – a transport step followed by a reaction step. The time step is determined by the Courant condition. This typically means a small time step, on the order of ~10s. However, this method reduces the numerical dispersion that can be introduced by an implicit approach.

First, concentration changes caused by advection and diffusion are solved first using an upwind scheme (eqn(S1)).

$$\frac{b_{i,j}^t\left(C_{i,j}^{t,trans}-C_{i,j}^t\right)}{\Delta t} = -\left(\frac{qx_{i+1/2,j}^t C_{i,j}^t - qx_{i-1/2,j}^t C_{i-1,j}^t}{\Delta x} + \frac{qy_{i,j+1/2}^t C_{i,j}^t - qy_{i,j-1/2}^t C_{i,j-1}^t}{\Delta y}\right) + Db_{i,j}^t\left(\frac{C_{i+1,j}^t + C_{i-1,j}^t - 2C_{i,j}^t}{\Delta x^2} + \frac{C_{i,j+1}^t + C_{i,j-1}^t - 2C_{i,j}^t}{\Delta y^2}\right) \qquad \text{(S1a)}$$

We note that this discretization, which was used for the simulations reported in the manuscript, is not conserved for mass because aperture b is spatially variable. A more rigorous discretization (eqn(S1b)) should be used, where $b_{i+\frac{1}{2},j}^t$ is the harmonic mean or geometric mean of the apertures of the two neighboring cells.

$$\frac{b_{i,j}^t\left(C_{i,j}^{t,trans}-C_{i,j}^t\right)}{\Delta t} = -\left(\frac{qx_{i+\frac{1}{2},j}^t C_{i,j}^t - qx_{i-\frac{1}{2},j}^t C_{i-1,j}^t}{\Delta x} + \frac{qy_{i,j+\frac{1}{2}}^t C_{i,j}^t - qy_{i,j-\frac{1}{2}}^t C_{i,j-1}^t}{\Delta y}\right) + D\left[\frac{b_{i+\frac{1}{2},j}^t\left(C_{i+1,j}^t - C_{i,j}^t\right)}{\Delta x^2} - \frac{b_{i-\frac{1}{2},j}^t\left(C_{i,j}^t - C_{i-1,j}^t\right)}{\Delta x^2} + \frac{b_{i,j+\frac{1}{2}}^t\left(C_{i,j+1}^t - C_{i,j}^t\right)}{\Delta y^2} - \frac{b_{i,j-\frac{1}{2}}^t\left(C_{i,j}^t - C_{i,j-1}^t\right)}{\Delta y^2}\right] \qquad \text{(S1b)}$$

To test this, for one of the simulation conditions of this study, we compared the results with and without this treatment of aperture. It was found that this treatment does not lead to measurable errors (Figure S1) in the simulations given the relatively small contribution of the lateral diffusion.

After transport, a speciation calculation is carried out to determine the concentrations of all aqueous species.

In the second step in the sequential non-iterative method, concentration changes due to reactions are calculated (eqn(S2)). The reaction rate is calculated based on updated concentrations of aqueous species after the transport step (with superscript trans) (eqn(S1)).

$$C_{i,j}^{t+\Delta t} = \frac{R_{calcite}\cdot\Delta t}{b_{i,j}^t} + C_{i,j}^{t,trans} \qquad \text{(S2)}$$

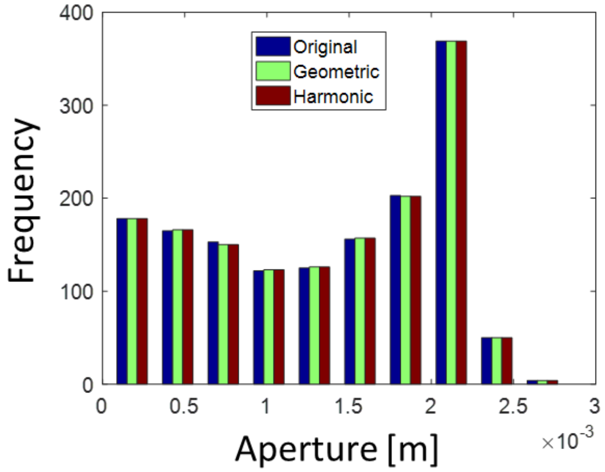

Figure S1. Histograms of fracture apertures from the simulations that use discretization scheme of eqn(S1a) (labelled as original), and the discretization of eqn(S1b) using geometric and harmonic means of neighbouring apertures. The simulations use the same conditions as the high P_{CO2} simulations, but the initial aperture field was further downsampled for computational efficiency.

The reaction rates are used to update the concentrations of TOTCa and TOTC at the end of the time step. The amount of reaction is then used to update *b* of the next time step according to mineral reactions using eqn(S3), in which $V_{calcite}$ is the molar volume of calcite.

$$b_{i,j}^{t+\Delta t} = R_{calcite} \cdot V_{calcite} \cdot \Delta t + b_{i,j}^{t} \tag{S3}$$

When significant change in the aperture occurs (every five minutes in the simulations), the flow field is updated. For the calculation of local pressure and volumetric flow rate, the Reynolds equation is discretized as follows:

$$\frac{T_{i-1/2,j}(P_{i-1,j}-P_{i,j})}{\mu\Delta x} - \frac{T_{i+1/2,j}(P_{i,j}-P_{i+1,j})}{\mu\Delta x} + \frac{T_{i,j-1/2}(P_{i,j-1}-P_{i,j})}{\mu\Delta y} - \frac{T_{i,j+1/2}(P_{i,j}-P_{i,j+1})}{\mu\Delta y} = 0 \tag{S4}$$

It describes steady state mass conservation for each grid cell. Please note that only the pressure contribution to the hydraulic head is considered.

The transmissivity at the boundary (e.g. $T_{i-1/2,j}$) is the harmonic mean of transmissivities of the two neighboring grid cells, and the transmissivity of each grid cell ($T_{i,j}$) is calculated from local aperture following the cubic law:

$$T_{i,j} = \frac{\Delta x b_{i,j}^3}{12} \ or \ \frac{\Delta y b_{i,j}^3}{12} \tag{S5}$$

2. Spatial correlation length of aperture field

The spatial correlation of the fracture aperture field was characterized by the semivariogram method (eqn(S6)). The semivariogram ($\gamma(h)$) with a lag of h defined as:

$$\gamma(h) = \frac{1}{2N(h)}\sum_{N(h)}\big(b(x+h) - b(x)\big)^2 \qquad \text{(S6)}$$

The semivariogram range (λ), which is the distance at which the semivariogram becomes independent of the lag value, defines the spatial correlation length of the apertures.

3. Potential sources of the discrepancy between the high P_{CO2} simulation and experiment

To investigate the potential impacts of the boundary layer diffusion limitation on reaction rate, we tested the approach used in *Hanna and Rajaram (1998)* and *Andre and Rajaram (2005),* where the lesser of the surface reaction rate and the diffusion reaction rate is used. The diffusion reaction rate is written as a function of only the calcium concentration, using the equilibrium calcium concentration ($C_{Ca,eq}$) and calcium concentration in the bulk ($C_{Ca,bulk}$). For the test simulations, unlike previous common practices where $C_{Ca,eq}$ is derived directly from the equilibrium constant, the equilibrium concentration used is derived from a batch reactor simulation using the influent composition as the initial fluid chemistry.

$$R_{diff} = \frac{ShD_m}{2b}\left(C_{Ca,eq} - C_{Ca,bulk}\right) \qquad \text{(S7)}$$

where D_m is the molecular diffusion coefficient, b is local aperture, and Sh is the Sherwood number and a value of 8 is used [*Szymczak and Ladd,* 2012].

The test was implemented in CrunchFlow [*Steefel et al.,* 2015]. For the test simulations, the conditions of the high P_{CO2} simulation was used. As a coarser mesh is used to reduce simulation time, the resulting dissolution pattern is more diffuse than the one shown in the manuscript. The increase of fracture aperture after 58 hours of reactive flow from the simulation with the diffusion limitation is smaller (Figure S2(a) and (b)), with an average aperture smaller by 19%, than that from the simulation without the diffusion limitation. However, the aperture fields with and without accounting for the diffusion limitation show similar spatial patterns. The evolution of the hydraulic aperture with respect to the fracture volume in the case with consideration of the diffusion limitation is also similar to that in the case without diffusion limitation (Figure S2(c)). These observations confirm that the

diffusion limitation across the fracture aperture can reduce the overall reaction but does not affect the dissolution patterns, i.e. channelization, significantly. Therefore, using the model to compare how variations in geochemical factors such as influent chemistry and mineral spatial distribution affect fracture channelization is justified.

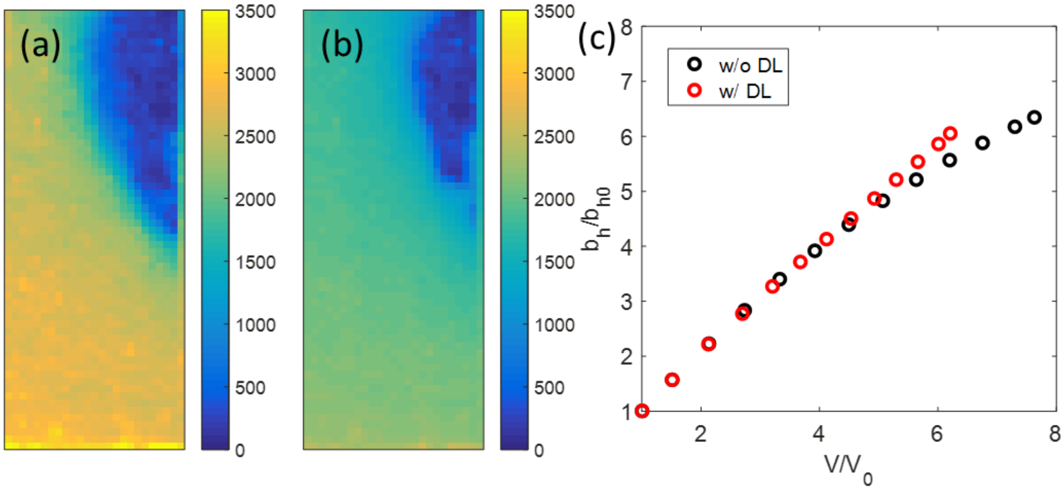

Figure S2. Aperture maps after 58 hours of reactive flow for the simulation (a) without and (b) with diffusion limitation of the boundary layer **across** the fracture aperture.

The differences in the spatial patterns of fracture channelization between the model results and the experimental data (Figure S3(a)) may be caused by the existence of local 'unreactive' zones suggested by close examination of the experimental data. The close-ups of the aperture map near the inlet (Figure S3(b)-(c)) show regions that were only slightly affected by the reactive fluid after 57.7 hours of flow. The cross-section from the segmented *xCT* image (Figure S3(d)) also indicates the persistent flow stricture at the inlet, which is not preserved in the 2D simulation results (Figure S3(e)). We speculate that these unreactive zones may be attributed to mineralogical heterogeneity such as impurities in the carbonate and the presence of different surface morphologies [*Levenson and Emmanuel*, 2013; *MacInnis and Brantley*, 1992]. Alternatively, there may be small-scale hydrodynamic heterogeneity, such as transverse dispersion within the porous rock [*Boon et al.*, 2016]. Note that the hydrodynamic heterogeneity here does not refer to flow perturbations below the grid resolution in the fracture plane. Even though channelization is very sensitive to such perturbations [*Szymczak and Ladd*, 2009; *Szymczak and Ladd*, 2012], in our study, the down-sampling of the aperture map and the relatively coarse resolution did not contribute significantly to the observed discrepancy, as the simulations with finer resolution (150 μm) generated similar dissolution patterns and effluent concentrations, decreasing from an initial value of 1460 mg/L to 1207 mg/L after 58 hours of simulation. Instead, as pointed out in previous study, the pore structures of the rock matrix may affect local flow and cause the fluid to bypass choking points in

the fracture [*Landry and Karpyn*, 2012]. Even though the Indiana Limestone is mineralogically homogeneous, it has been pointed out that its pore structure is highly heterogeneous [*Selvadurai and Selvadurai*, 2014].

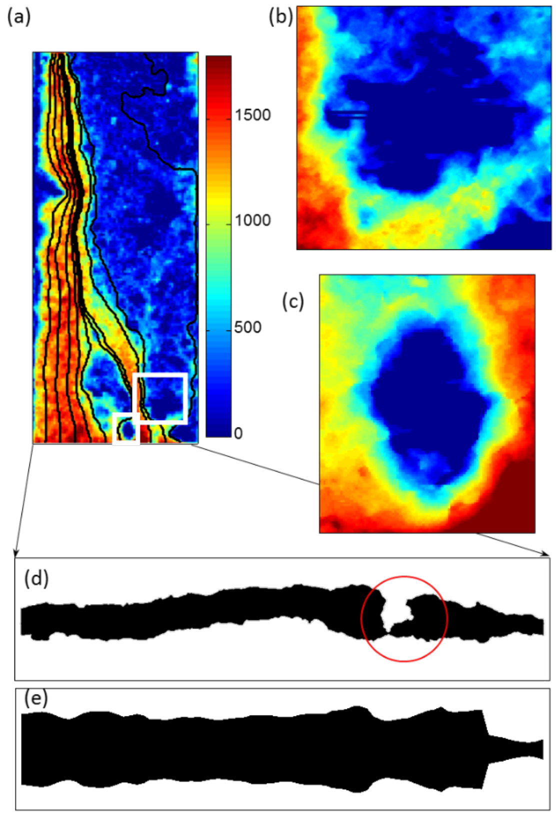

Figure S3. (a) Aperture map and streamlines of the reacted fracture of the high P_{CO2} experiment after 57.7 hours. (b)-(c) Close-ups of the unreacted zones shown in the white boxes in (a). (d) Cross-section from the segmented xCT image highlighting the unreacted zones near the inlet, and (e) corresponding cross-section from the simulation, depicted with the assumption of symmetry of the two fracture surfaces.

4. Simulation results for the G2 fracture geometry

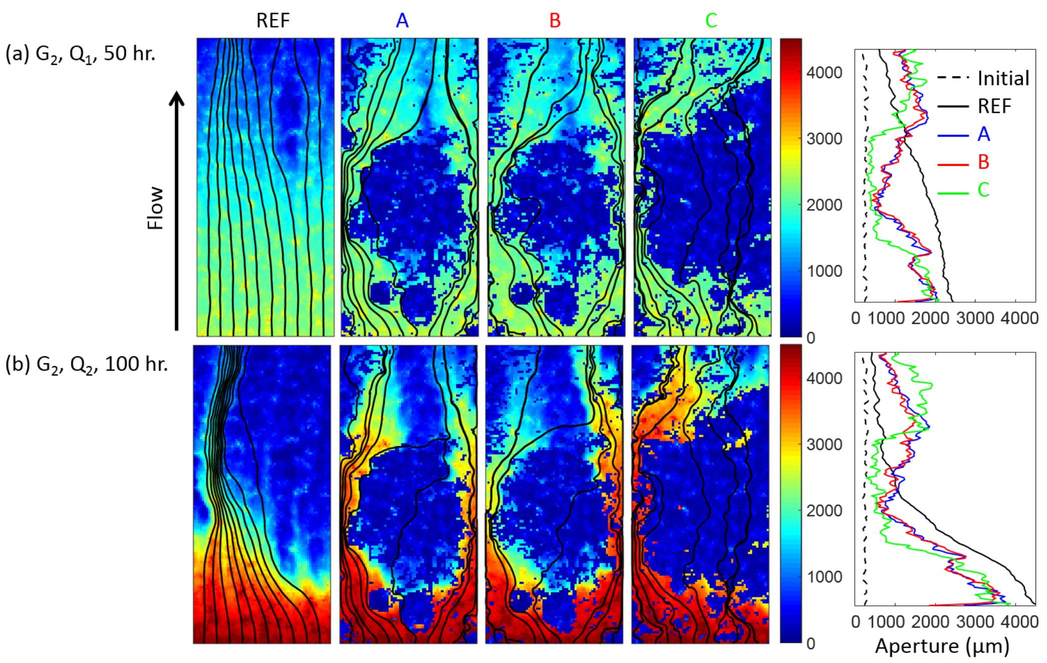

Figure S4. Aperture maps with streamlines and horizontally-averaged aperture along the flow direction (last column) from the simulations of (a) fracture G_2 at flow rate Q_1 after 50 hours of reaction and (b) fracture G_2 at flow rate Q_2 after 100 hours of reaction, i.e. the same total flow.

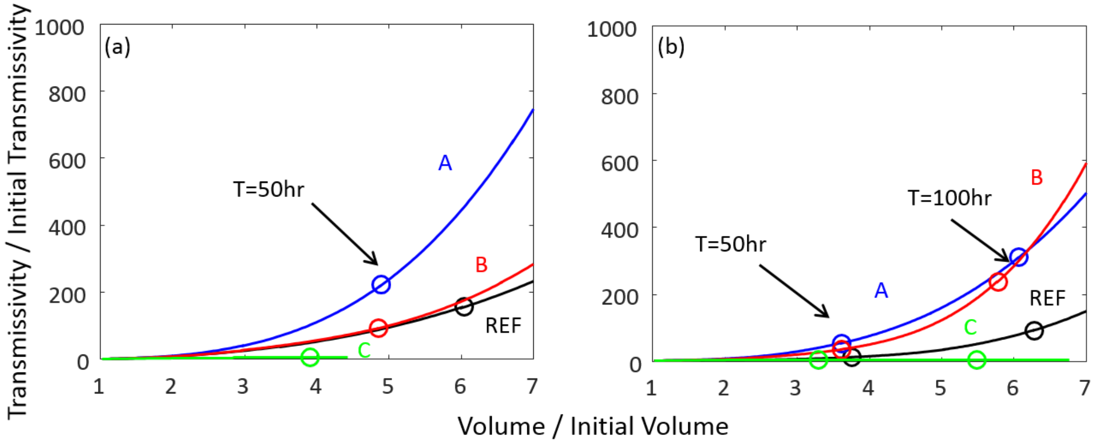

Figure S5. Transmissivity increase in relation to fracture volume increase for different mineral spatial patterns and flow rates. These simulation results are for fractures with geometry G_2. The circles highlight the prediction values at hour 50 for flow rate Q_1, and hour 100 for flow rate Q_2.

REFERENCES

Andre, B. J. and H. Rajaram (2005), Dissolution of limestone fractures by cooling waters: Early development of hypogene karst systems, *Water Resour. Res., 41*, n/a-n/a.

Boon, M., B. Bijeljic, B. Niu, and S. Krevor (2016), Observations of 3-D transverse dispersion and dilution in natural consolidated rock by X-ray tomography, *Advances in Water Resources, 96*, 266-281, doi: http://dx.doi.org/10.1016/j.advwatres.2016.07.020.

Hanna, R. B. and H. Rajaram (1998), Influence of aperture variability on dissolutional growth of fissures in Karst Formations, *Water Resour. Res., 34*, 2843-2853.

Landry, C. J. and Z. T. Karpyn (2012), Single-phase lattice Boltzmann simulations of pore-scale flow in fractured permeable media, *International Journal of Oil Gas and Coal Technology, 5*, 182-206.

Levenson, Y. and S. Emmanuel (2013), Pore-scale heterogeneous reaction rates on a dissolving limestone surface, *Geochim. Cosmochim. Acta, 119*, 188-197.

MacInnis, I. N. and S. L. Brantley (1992), The role of dislocations and surface morphology in calcite dissolution, *Geochim. Cosmochim. Acta, 56*(3), 1113-1126, doi: http://dx.doi.org/10.1016/0016-7037(92)90049-O.

Selvadurai, P. A. and A. P. S. Selvadurai (2014), On the effective permeability of a heterogeneous porous medium: the role of the geometric mean, *Philos. Mag., 94*, 2318-2338.

Steefel, C. I. et al. (2015), Reactive transport codes for subsurface environmental simulation, *Computational Geosciences, 19*, 445-478.

Steefel, C. I. and K. T. B. MacQuarrie (1996), Approaches to modeling of reactive transport in porous media, *Reactive Transport in Porous Media, 34*, 83-129.

Szymczak, P. and A. Ladd (2009), Wormhole formation in dissolving fractures, *Journal of geophysical research, 114*, B06203.

Szymczak, P. and A. J. C. Ladd (2012), Reactive-infiltration instabilities in rocks: Fracture dissolution, *J. Fluid Mech., 702*, 239-264.
